# Development and growth trends in angiotensin II‐induced murine dissecting abdominal aortic aneurysms

**DOI:** 10.14814/phy2.13668

**Published:** 2018-04-25

**Authors:** Amelia R. Adelsperger, Evan H. Phillips, Hilda S. Ibriga, Bruce A. Craig, Linden A. Green, Michael P. Murphy, Craig J. Goergen

**Affiliations:** ^1^ Weldon School of Biomedical Engineering Purdue University West Lafayette Indiana; ^2^ Department of Statistics Purdue University West Lafayette Indiana; ^3^ IU Health Center for Aortic Disease/Department of Surgery Indiana University School of Medicine Indianapolis Indiana; ^4^ Richard L. Roudebush VA Medical Center Indianapolis Indiana

**Keywords:** aneurysm, angiotensin, aorta, ultrasound

## Abstract

Abdominal aortic aneurysms are pathological dilations that can suddenly rupture, causing more than 15,000 deaths in the U.S. annually. Current treatment focuses on observation until an aneurysm's size warrants surgical intervention. Thus, there is a need for therapeutic intervention to inhibit growth of smaller aneurysms. An experimental aneurysm model that infuses angiotensin II into apolipoprotein E‐deficient mice is widely used to investigate underlying pathological mechanisms and potential therapeutics, but this model has two caveats: (1) aneurysms do not always form, and (2) aneurysm severity and growth is inconsistent among animals. Here we use high‐frequency ultrasound to collect data from angiotensin II‐induced aneurysms to develop prediction models of both aneurysm formation and growth. Baseline measurements of aortic diameter, volume/length, and strain were used with animal mass and age in a quadratic discriminant analysis and logistic regression to build two statistical models to predict disease status. Longitudinal ultrasound data were also acquired from mice with aneurysms to quantify aneurysm diameter, circumferential strain, blood flow velocity, aneurysm volume/length, and thrombus and open‐false lumen volumes over 28 days. Measurements taken at aneurysm diagnosis were used with branching artery information to produce a multiple linear regression model to predict final aneurysm volume/length. All three statistical models could be useful in future aneurysm therapeutic studies to better delineate the effects of preventative and suppressive treatments from normal variations in the angiotensin II aneurysm model.

## Introduction

Abdominal aortic aneurysms (AAAs) are localized dilations of the abdominal aorta that are often asymptomatic, and lead to over 150,000 deaths globally in 2013 due to sudden aortic rupture and internal hemorrhage (Hirsch et al. 2005; Wang et al. 2015). The causes of initial development and subsequent growth of aneurysms are not fully understood and are a primary interest within the field of cardiovascular research. In humans, aneurysms with a diameter greater than roughly 5 cm have a higher risk of rupture and are therefore recommended for intervention (Brewster et al. 2003). The two current surgical procedures are endovascular stent graft placement and open repair, both of which are associated with complications (Sweeting et al. 2012). Thus, there is a need for medical treatments to delay the growth of aneurysms and consequently delay or eliminate the need for surgical intervention (Davis et al. 2014). Although therapy development efforts often show promising early results, translating findings from small animal studies into humans remains challenging (Davis et al. 2014).

To better understand the human disease and evaluate a range of experimental treatments, there are several commonly used murine AAA models. Two of the most popular models include: (1) application of porcine pancreatic elastase (Pyo et al. 2000; Bhamidipati et al. 2012), and (2) induction of dissecting AAA in hyperlipidemic mice, commonly apolipoprotein E‐deficient (apoE^−/−^) or low density lipoprotein receptor deficient (LDLR^−/−^), that are infused with angiotensin II (AngII) (Daugherty and Cassis 1999; Daugherty et al. 2000; Manning et al. 2002; Saraff et al. 2003). While there is not an exact replica of human AAA in mice, both of these murine models resemble different facets of the human condition (Daugherty and Cassis 2004). The elastase procedure induces aortic enlargement 100% of the time, but involves a complicated abdominal surgery that is often associated with a high mortality rate. The AngII‐induced dissecting AAA model, while not like true human aneurysms that form in the infrarenal aorta, is commonly used due to ease of creation and its ability to mimic male predisposition, hyperlipidemia, and hypertension (Daugherty et al. 2000; Saraff et al. 2003; Daugherty and Cassis 2004; Cao et al. 2010). This model creates dissecting suprarenal saccular aneurysms, rather than infrarenal fusiform expansions (Daugherty and Cassis 2004; Goergen et al. 2010, 2011), (typically 3 to 10 days after pump implantation) (Daugherty and Cassis 2004; Cao et al. 2010). Unfortunately, a meta‐analysis of 194 different publications showed that the AngII model only creates dissecting AAAs in roughly 60% of animals, with death due to spontaneous aortic rupture in another 20% and the remaining 20% of mice experiencing neither event (Trachet et al. 2015a). This variability is a major limitation in the AngII model when evaluating experimental therapeutics.

In order to quantify aneurysm progression in these models, noninvasive imaging modalities such as ultrasound (US), magnetic resonance (MR), and computed tomography (CT) have been utilized to characterize aortic expansion in vivo (Ramaswamy et al. 2013). High‐frequency US, specifically, has been used to characterize murine AAAs for more than 10 years (Martin‐McNulty et al. 2005). This type of system can quickly and accurately collect real‐time imaging data with high resolution and without the need for ionizing radiation or contrast agents (Martin‐McNulty et al. 2005; Ramaswamy et al. 2013; Phillips et al. 2015). In general, longitudinal studies that utilize in vivo US allow for the assessment of vessel diameter, volume, motion, strain, and blood flow velocities from multiple time points throughout dissecting AAA disease progression for each animal (Trachet et al. 2015a).

A caveat with testing therapeutics in the AngII model is that aneurysm formation is not always consistent (Cao et al. 2010; Trachet et al. 2015a), and the dissecting aneurysms vary in size and severity (Prins et al. 2014). Unlike surgical experimental models of AAAs, the AngII model can cause a dissecting AAA, aortic rupture, or no breakage of aortic elastin and a seemingly normal vessel (Trachet et al. 2015a). In this study, we used baseline measurements acquired with high‐frequency US for 94 male apoE^−/−^ mice implanted with osmotic mini‐pumps filled with AngII. The purpose of this study was to identify potential biomechanical predictors that predispose animals to the development of a dissecting AAA, aortic rupture, or neither. Here we present results of using baseline measurements to predict if an animal's aorta will become diseased or stay nondiseased, using two statistical models. We also present results from longitudinal data to predict trends in growth once an aneurysm has formed. Overall, these indicators of aneurysm formation and growth will aid in future treatment efficacy studies by more clearly delineating risk of aneurysm formation and expansion on a mouse‐specific basis.

## Materials and Methods

### Animal care and maintenance

Male apoE^−/−^ mice (*n* = 94, 29.0 ± 3.5 g, 15.7 ± 8.0 weeks old; B6.129P2‐Apoe^tm1Unc^/J) from The Jackson Laboratory (Bar Harbor, ME) were used in this study. We purposely used animals with a large range in age to strengthen our prediction models and to replicate diverse ages found in literature. The animals, housed in groups of five, were given free access to standard rodent chow and water. Each animal's mass was measured prior to the start of the study and tracked on subsequent surgery and imaging days to ensure wellness. The Purdue Animal Care and Use Committee approved all procedures.

### Ultrasound image collection

#### Animal preparation

All apoE^−/−^ mice underwent US imaging prior to surgery and on days 7, 14, 21, and 28 postimplantation. For all imaging, mice were knocked‐down with 3% isoflurane (Isothesia, Henry Schein Animal Health, Dublin, OH) in 1.5 L/min of medical air and switched to 1.5–2% isoflurane to maintain unconsciousness. The animals were placed supine on a heated stage, eye lubricant was applied, and depilatory cream (Nair, Church & Dwight Co., Inc. Ewing, NJ) was used on the ventral abdomen to remove hair. Warmed US transmission gel was applied to the skin prior to placement of the US probe. Heart and respiratory rate as well as internal body temperature were monitored throughout imaging with stage electrodes and a rectal probe, respectively.

#### Image collection

In order to detect and monitor the development and growth of a dissecting AAA, US images were collected using Vevo2100 (FUJIFILM Visual Sonics, Toronto, ON, Canada) and the MS550D, 25–55 MHz probe with a center frequency of 40 MHz. Longitudinal, two‐dimensional brightness mode (B‐mode), ECG‐gated kHz visualization (EKV), and color Doppler cine loop images were collected suprarenally, as well as motion mode (M‐mode) and pulsed wave (PW) Doppler images at proximal, middle, and distal suprarenal locations. The stage's angle was adjusted during PW image collection so the cursor was parallel to the vessel walls to ensure accurate velocity measurements. We also collected transverse, two‐dimensional B‐mode and EKV images at proximal, middle, and distal suprarenal locations. Finally, three‐dimensional images were collected over a distance of 15–20 mm using cardiac and respiratory gating with a step size of 0.19 mm.

### Angiotensin II pump implantation procedure

#### Pump preparation

To induce aneurysms, all mice were implanted with osmotic mini‐pumps (ALZET Model 2004, DURECT Corporation, Cupertino, CA) filled with AngII (Bachem, Torrance, CA). We used 0.9% sodium chloride to dilute the AngII to an infusion rate of 1000 ng/kg/min for 28 days. Doses were unique to each mouse based on animal mass on the day of pump preparation. Once filled with the AngII solution, pumps were placed in saline and warmed to 37°C for 12–24 h prior to implantation.

#### Animal procedure

Mice were first anesthetized with 3% isoflurane in 1.5 L/min of medical grade air and switched to 2% isoflurane to maintain anesthetic depth. A toe pinch ensured the animal was unconscious. Sterile eye lubricant was applied to each eye and depilatory cream was used to remove hair on the left, upper dorsal quadrant of the mouse. Sterile techniques were used to implant the pump subcutaneously into the back of the mouse. Suture clips (Reflex 7, CellPoint Scientific, Inc., Gaithersburg, MD) were used to close the wound and antibiotic ointment (Neosporin, Johnson & Johnson, Skillman, NJ) was applied to aid wound healing. Buprenorphine (0.1 mL of 0.03 mg/mL strength; Burprenex Injection, Reckitt Benckiser Pharmaceuticals, Inc., Richmond, VA) was given at time of surgery to alleviate discomfort.

### Ultrasound image analysis

#### Overview of analysis

US images were analyzed for all mice at baseline and on days 7, 14, 21, and 28 using Vevo2100 software (VevoLAB, FUJIFILM Visual Sonics). Longitudinal 2D B‐modes, longitudinal 2D M‐mode images, PW Doppler, and 3D B‐modes allowed us to measure maximum effective diameter, circumferential strain, centerline blood velocity, and true and false lumen volumes, as well as volumes for the open‐false lumens and thrombus within the dissection. All 2D and PW measurements were made in quintuplicate and averaged to reduce error. Dissecting AAAs could be identified with US by their increase in diameter, wall thickening and stiffening, changes in blood flow velocity, and presence of a dissection. The lesions were noticeable and quantifiable to the students who collected the images who were highly trained and experienced with this AAA model and imaging modality.

#### Two‐dimensional analysis

**Figure 1:**
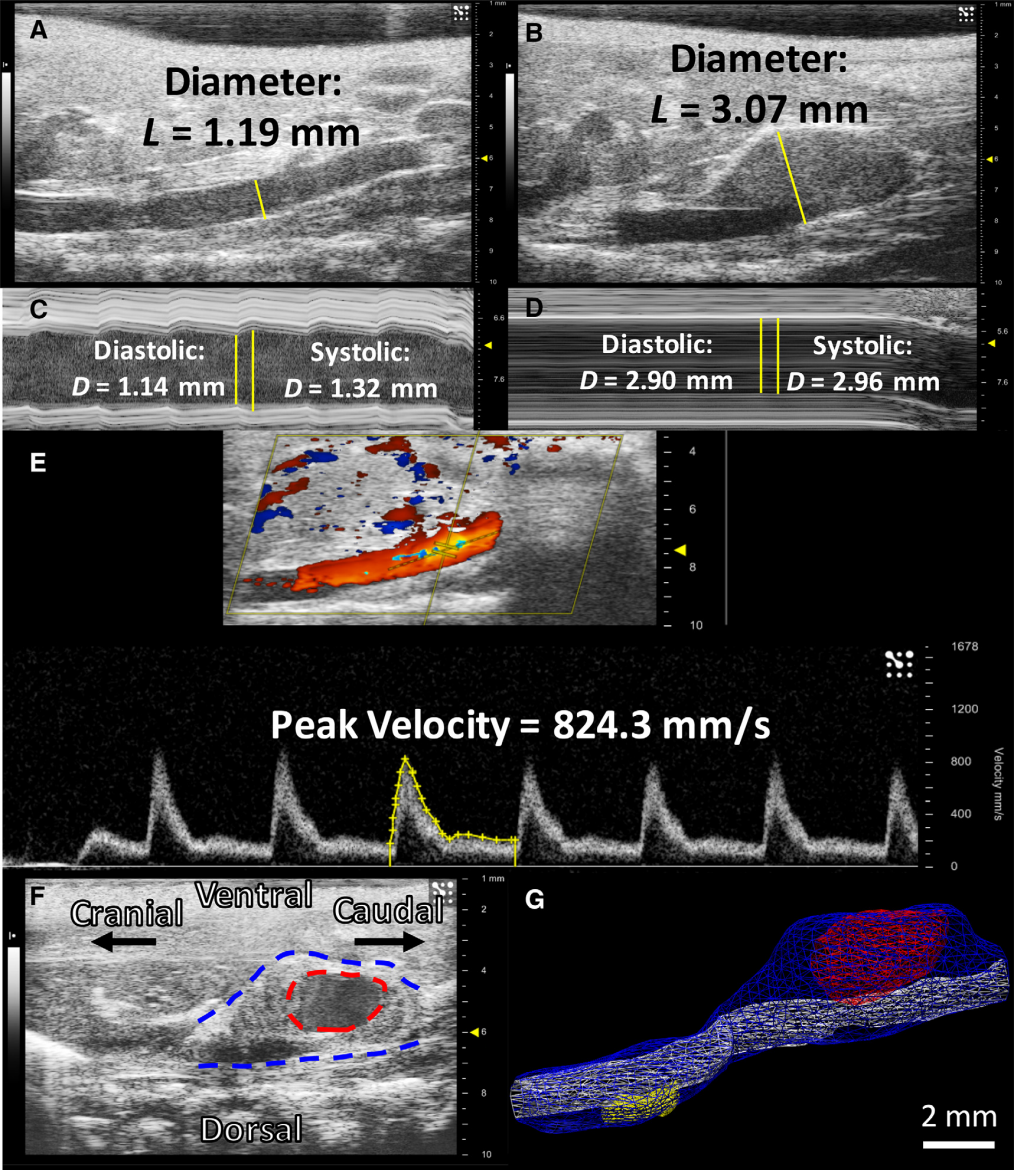
Sample 2D and 3D ultrasound images and analysis. 2D B‐mode images shown with example linear measurements placed perpendicular to the vessel walls in healthy (A) and aneurysmal (B) mice. 2D M‐mode images show cursor placement and example systolic and diastolic measurement tracings in a healthy (C) and diseased (D) aorta. The dotted line cursor for PW images runs parallel to the aorta, and a sample velocity waveform tracing is shown in yellow (E). A 2D long‐axis B‐mode image of an aneurysm is shown on the left (F) with a volumetric representation of this aneurysm shown on the right (G). The blue surface is the overall AAA volume, the white surface is the true lumen volume, the red represents the open‐false lumen near the renal arteries, and the yellow is a closed lumen from a more cranial lesion. The hypoechoic region on the B‐mode image confirms the open‐false lumen volume depicted in (G; dotted red line). This volumetric acquisition further demonstrates the benefits of 3D imaging for complex aortic dissections with multiple lesions.

Maximum outer diameters were measured with longitudinal B‐mode images using a linear measurement tool in VevoLAB. Although some groups suggest short‐axis B‐mode images are best for measuring diameter, this can also give incorrect measurements as it does not account for the natural curvature of the suprarenal murine aorta. In healthy and diseased vessels, systolic and diastolic diameter measurements were taken in the largest portion of the suprarenal aorta or aneurysm and angled to be perpendicular to the vessel walls (Fig. 1A and B). Mean diameter (*D*) was found using equation (1) .

(1) $$D_{\mathrm{mean}}=\left(\frac{1}{3}\left(D_{\mathrm{systolic}}-D_{\mathrm{diastolic}}\right)\right)+D_{\mathrm{diastolic}}$$

Longitudinal 2D M‐mode images were used to determine systolic and diastolic diameters in the middle of the suprarenal aorta or the largest portion of the aneurysm (Fig. 1C and D). These measurements were used to calculate the circumferential component of the Green‐Lagrange strain tensor using equation (2).

(2) $$\text{circumferential strain}=0.5\times \left({\left(\frac{D_{\mathrm{systolic}}}{D_{\mathrm{diastolic}}}\right)}^{2}-1\right)$$

We used PW Doppler images taken in the middle of the suprarenal aorta to determine peak and mean blood flow velocities (Fig. 1E). Velocity waveforms were manually traced to calculate the peak velocity, mean velocity, and velocity time integral (VTI).

#### Three‐dimensional analysis

We utilized 3D B‐mode images to manually segment overall volume in nondiseased aneurysms and overall, true lumen, false lumen, thrombus, and open‐false lumen volumes in aneurysmal vessels. The 3D measurements we acquire also provide another method for measuring the aorta without settling for 2D diameters. Consistent starting points and lengths were used across different study days for each mouse and were calculated across the acquired z‐slices. Starting locations for the volumetric contours were based off the day 28 images and were placed with respect to the right renal artery. Mouse‐specific dissecting AAA lengths were also calculated from day 28 images and were kept consistent for all previous days, including baseline. Total lengths were used in every per length calculation. The starting point for each healthy vessel was at the right renal artery, and contours were drawn up the length of the suprarenal aortic curve toward the thoracic aorta. For mice with dissecting AAA, overall, true lumen, and open‐false lumen volumes were measured individually. False lumen volumes were calculated from the difference between overall and true lumen volumes, and thrombus volumes were calculated from the difference between false lumen and open‐false lumen volumes. Open‐false lumen volumes could be visualized by their hypoechoic appearance and by cross‐referencing longitudinal and transverse B‐mode, M‐mode, and EKV images (Fig. 1F and G). Additionally, we were able to visualize the superior mesenteric (SMA) and celiac arteries, providing us with the ability to quantify the number of branching vessels (0,1, or 2) directly connected to the aneurysm.

### Animal sacrifice and dissection

On day 28 of the study, all nonruptured animals were euthanized and their tissues were removed. After being anesthetized with 3% isoflurane in 1.5 L/min of medical grade air, we performed bilateral thoracotomy. The whole aorta, anchored by the heart, the kidneys, and surrounding back tissue, was excised and placed in 10% formalin for 24 h.

### Histology

**Figure 2:**
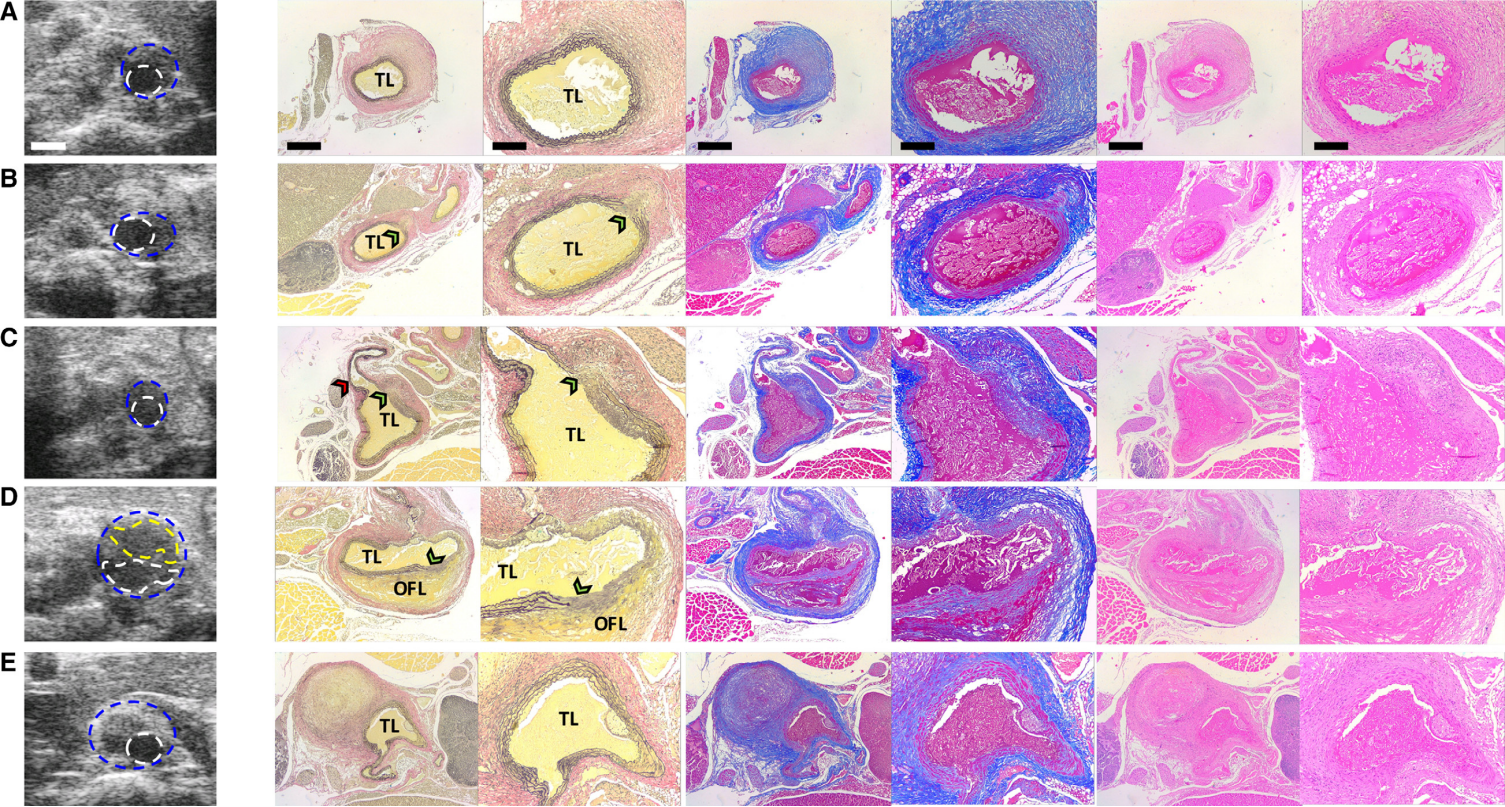
Sample histology taken at day 28 post‐AngII infusion matches well with US imaging data. 2D short‐axis US images near the proximal end of AAAs are shown with corresponding histology (from left to right: VVG, MTC and H&E stains at low 4× and high 10× magnification) in 5 different animals (A–E). VVG stain shows evidence of elastin degradation and aortic dissection (green arrowheads in B, C, and D) and MTC stain highlights adventitial collagen remodeling (blue staining in A–E). An open‐false lumen (OFL) is confirmed by histology for one lesion (D) and atherosclerotic plaque for three lesions (C, D, and E). All lesions (except E) show evidence by US and histology of leftward expansion. A small rightward expanding lesion immediately below the diaphragm (E) is identified proximal to a larger leftward‐expanding lesion in the same animal (see Figure 1G). Blue line = outer vessel wall; White line = inner vessel wall; Yellow line = outline of open‐false lumen; TL = true lumen; Red arrowhead = celiac artery. Scale bars: 1 mm (US), 500 μm (low magnification histology), and 200 μm (high magnification histology).

Fixed tissues were placed in 70% ethanol and processed for paraffin histology as per standard protocols. From the cranial end of the suprarenal aorta, we collected 10‐μm‐thick cross‐sectional slices of the aorta and surrounding tissues and stained adjacent sections to assess morphology (hematoxylin and eosin) and vessel wall proteins (Verhoeff Van‐Gieson for elastin and Masson's Trichrome for collagen). We acquired images at 4× and 10× magnification (DM750 equipped with ICC50W camera, Leica Microsystems) and compared these to the same short‐axis views of the three‐dimensional US scans (Fig. 2).

### Statistical methods

To determine significant differences among groups or time points, one‐way ANOVAs with Tukey post hoc tests were run using Minitab statistical software (version 17, Minitab, State College, PA). We considered *P*‐values of 0.05 or less to be significant. All other statistical models were run using Statistical Analysis System (version 9.4, SAS Institute Inc., Cary, 115 NC).

#### Logistic regression prediction

A logistic regression analysis was conducted to predict the aneurysm status (nondiseased; diseased) for mice using baseline mass, diameter, age, strain, volume, and peak blood flow velocity as predictors for 94 mice. Descriptive statistics for these animals are included in Table 1 below. A backward variable selection method with an exit alpha level of 0.4 was implemented for variable prescreening, which removed peak velocity from the list of important predictors for this logistic regression model. The remaining predictors (mass, diameter, age, volume, and strain) were used in the final predictive model. We utilized leave‐one‐out cross validation methods for this model to estimate the misclassification error rate (Hastie et al. 2009). This method involves fitting the logistic regression for all animals except one. The fitted model was then used to predict the disease status of the one mouse left out. The algorithm was repeated to predict disease status for all 93 other mice. The prediction success was computed by dividing the number of correct predictions obtained by the total number of mice.

**Table 1:** Descriptive statistics for animals used in disease status predictive models

Predictor variables	*N*	Minimum	Maximum	Mean	Std. deviation
Mass	94	21.8	42.9	28.9	3.48
Age	94	9.7	45.4	15.6	7.97
Mean diameter (mm)	94	0.696	1.387	1.011	0.113
Peak blood flow velocity (mm/sec)	94	381	1144	754	182
Circumferential strain (mm/mm)	94	0.086	0.294	0.196	0.043
Volume/length (mm^2^)	94	0.408	1.200	0.811	0.147

#### Quadratic discriminant analysis prediction

A quadratic discriminant analysis with baseline variables mass, age, peak velocity, diameter, volume and strain were used to predict the disease status for the 94 mice. Homogeneity and normality tests were done on the data to confirm that a discriminant analysis could be performed accurately. The same stepwise variable selection method was implemented with a variable entry alpha level of 0.4 for screening out variables that contribute marginally to the quadratic discriminant function. We again utilized leave‐one‐out cross‐validation methods to estimate the misclassification error rate of this model (Hastie et al. 2009). The model percentage prediction success was then computed by counting the number of correct predictions divided by the total number of predictions.

#### Multiple linear regression prediction

To determine growth trends in animals with dissecting AAA, we generated a linear regression model to predict final (day 28) volume/length for each animal based on US measurements taken on the first day an aneurysm was observed (diagnosis day). Six diagnosis day measurements were used to predict final aneurysm volume/length: (1) diameter, (2) strain, (3) peak velocity, (4) open‐false lumen (OFL) volume, (5) thrombus volume, and (6) the number of branches feeding into the aneurysm. Interaction terms were also investigated to achieve a clearer relationship between variables in the model. Both a full model using 3D measurements and a simplified model using only 2D measurements were developed and analyzed.

## Results

### Baseline aortic measurements from high‐frequency ultrasound

**Figure 3:**
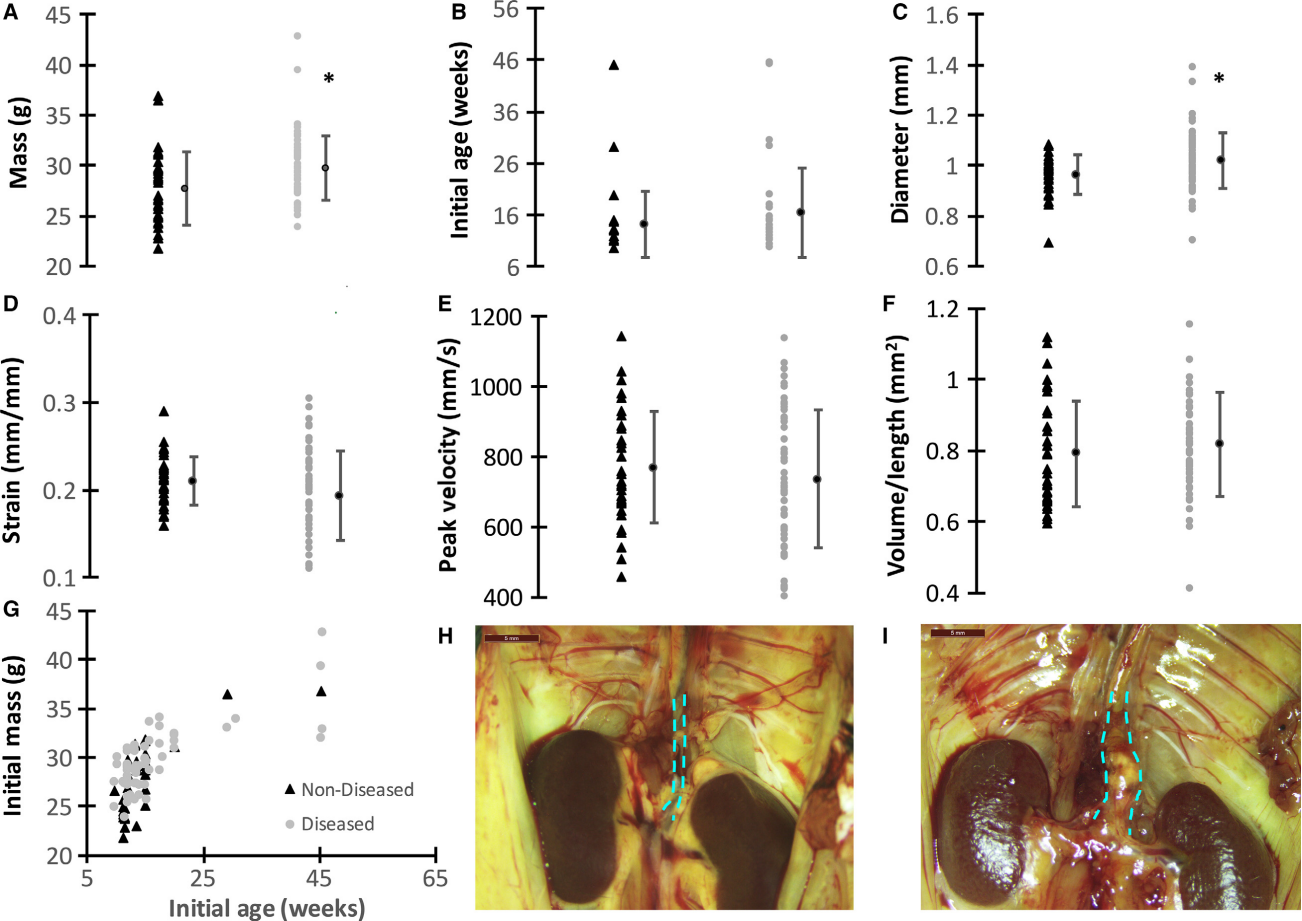
Baseline suprarenal aortic US measurements compared between groups. Both nondiseased (black triangle) and diseased (gray circles) are plotted from individual animals with means for each group shown as black circles (A–F). No significant differences were observed between groups except for body mass and initial mean diameter (*P ≤ 0.01). As expected, body mass was correlated with age for both the nondiseased and diseased groups (G). Example nondiseased (H) and aneurysmal (I) suprarenal aortas are shown at day 28 after sacrifice. Dashed cyan lines highlight the suprarenal abdominal aorta where AngII‐induced aneurysms form immediately proximal to the renal arteries. The aneurysmal aorta shows a dissection bulging to the animal's left. Scale bars: 5 mm. These plots represent the negligible differences between individual metrics for diseased and nondiseased animals, prompting the need for a statistical model to better delineate between the groups.

Of the 94 mice we used, 16 experienced aortic rupture, 43 formed an aneurysm, and 35 remained healthy after AngII infusion. For our dissecting AAA prediction model, mice that either developed an aneurysm or experienced aortic rupture were averaged together and referred to as the diseased group since we did not have enough mice to separate the data into two groups. Figure 3 shows the spread of these values with averages (B‐G) as well as initial mass and age plotted against each other for both groups (G). Baseline US measurements of mean age, mass, diameter, circumferential strain, peak blood flow velocity and volume/length in each animal are reported in Table 2 with further examples in Figure 1. None of the individual metrics was statistically different between the two groups except initial mass and mean diameter was slightly greater in the diseased group (*P* < 0.05).

**Table 2:** Baseline ultrasound metrics for animals that eventually developed aneurysms or experienced aortic rupture (diseased) and for those that did not (non‐diseased)

Variable	Non‐Diseased	Diseased
n	35	59
Age (weeks)	14.3 ± 6.3	16.5 ± 8.8
Mass (g)	27.7 ± 3.6	29.7 ± 3.2*
Mean diameter (mm)	0.964 ± 0.08	1.02 ± 0.11*
Circumferential strain (mm/mm)	0.211 ± 0.03	0.193 ± 0.05
Peak blood flow velocity (mm/sec)	768 ± 159	736 ± 196
Volume/length (mm^2^)	0.791 ± 0.15	0.817 ± 0.15

*Statistical differences (*P* < 0.05) shown as * when compared to the non‐diseased group. Data shown as mean ± SD.

### Changes in diameter, peak velocity, strain, and volume over 28 days

Longitudinal image data were also analyzed for 43 of the 94 animals described above (16 with dissecting AAAs and 27 nondiseased). Longitudinal data could not be collected from animals that experienced aortic rupture after pump implantation as these animals spontaneously died within the first 14 days after pump implantation. The remaining 35 mice could not be analyzed longitudinally because they were part of other studies with different endpoints and were included retrospectively into this study. For the nondiseased group between day 0 and day 28 (Table 3), diameter and volume/length were significantly increased (*P* < 0.001), whereas the circumferential strain and peak flow velocity were significantly decreased (*P* < 0.001). Additionally, peak flow velocity (*P* < 0.01) and the other three metrics were statistically greater (*P* < 0.001) for mice in the dissecting AAA group between day 0 and day 28. Figure 3H,I illustrates the difference between two suprarenal aortas with and without an aneurysm on day 28 at sacrifice. Not surprisingly, when comparing nondiseased and dissecting AAA groups at day 28, the aortic diameter, peak flow velocity, and volume/length were all found to be statistically greater and strain was found to be statistically smaller in the diseased group (*P* < 0.001). No significant differences were found in the US measurements, initial age, or body mass, when we compared day 0 data between groups.

**Table 3:** Longitudinal ultrasound metrics compared between groups and time points

Variable	Nondiseased		AAA	
	Day 0	Day 28	Day 0	Day 28
*n*	27	N/A	16	N/A
Initial age (weeks)	15.0 ± 7.1	N/A	16.9 ± 8.9	N/A
Initial mass (g)	27.9 ± 4.0	N/A	29.5 ± 2.5	N/A
Mean diameter (mm)	0.968 ± 0.08	1.175 ± 0.17^	1.00 ± 0.07	2.215 ± 0.51* ^,^ ^
Circumferential strain (mm/mm)	0.212 ± 0.03	0.141 ± 0.03^	0.200 ± 0.03	0.039 ± 0.01* ^,^ ^
Peak blood flow velocity (mm/sec)	785 ± 169	521 ± 176^	825 ± 185	1188 ± 458* ^,^ ^
Volume/Length (mm^2^)	0.775 ± 0.12	1.180 ± 0.26^	0.783 ± 0.08	2.759 ± 1.15* ^,^ ^

*Statistical differences (*P* < 0.05) shown as * when compared with the nondiseased group, ^^^ when compared with day 0 within same group. Data shown as mean ± SD.

### Changes in total, thrombus, and open‐false lumen volumes

**Figure 4:**
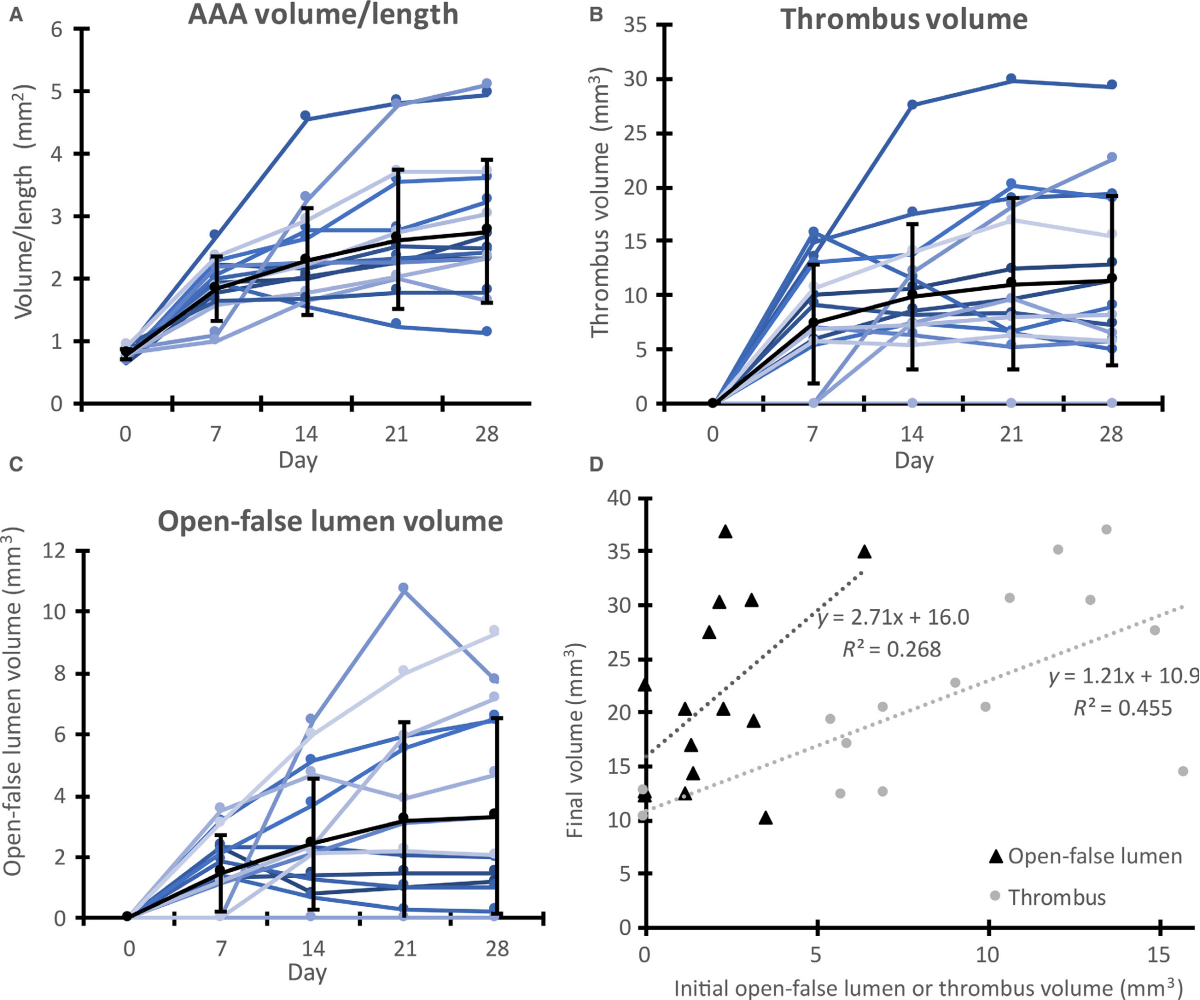
Aneurysm growth from volumetric analysis of the total AAA, open‐false lumen, and intramural thrombus. Volume data are shown for days 0 through 28 of total dissecting AAA volume/length (A) and also for thrombus volume (B) and open‐false lumen volume (C) within the aneurysmal false lumen. Each line represents an individual mouse. Day 0 represents measurements at baseline, prior to AngII infusion. Black lines show means ± SD. Initial (or diagnosis day) open‐false lumen and thrombus average volumes show a positive trend when plotted with respect to final dissecting AAA volume (D). These plots show how the composition of an aneurysm may influence the growth of a dissecting AAA over time.

Total volume, open‐false lumen volume, and thrombus volume were quantified on days 0, 7, 14, 21, and 28 for a subset of 16 mice with dissecting AAAs (Fig. 4). Animals had thrombus, an open‐false lumen, or both within the aneurysm. Here we define the false lumen as the intramural volume outside the true lumen that expands with dissecting AAA development, and we define the open‐false lumen as the open channel within the false lumen. As expected, larger thrombus and open‐false lumen volumes were generally associated with larger total dissecting AAA volumes. The thrombus and open‐false lumen volumes from the first day the aneurysm was observed (diagnosis day) were plotted against the final aneurysm volume to characterize the relationship between initial and final dissecting AAA size (Fig. 4D). Although there was not a strong linear relationship (open‐false lumen *R* ^2^: 0.268, thrombus *R* ^2^: 0.455), the general trend shows a positive relationship between open‐false lumen or thrombus volumes and final dissecting AAA volume, with slopes of 2.71 and 1.21 mm^3^/day, respectively.

### Effects of branching arteries on AAA growth

**Figure 5:**
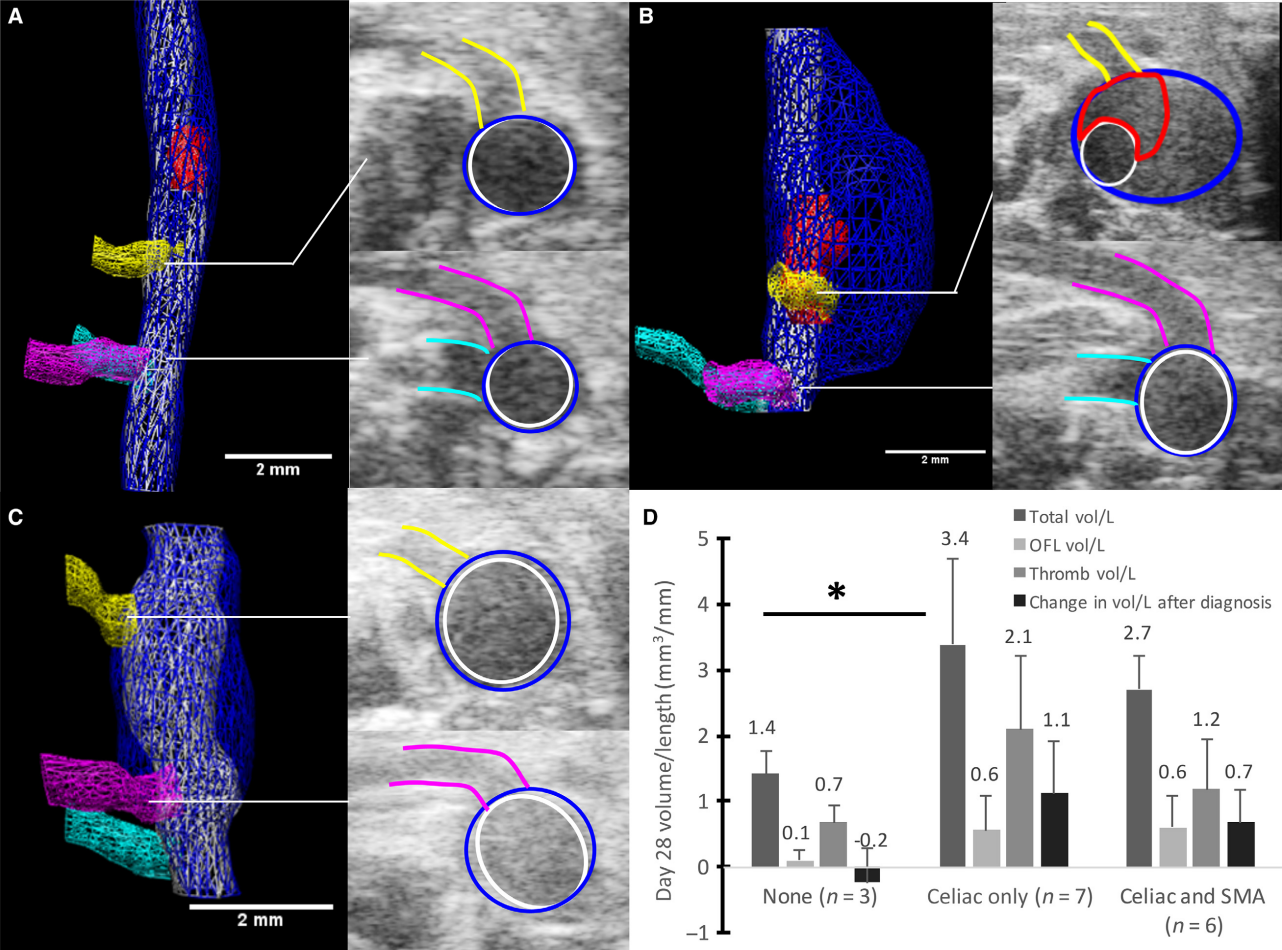
Effects of branching arteries on dissecting AAA growth. Volume segmentations of mice with no branching arteries (A), only the celiac artery branching into the expanded region of the vessel (B), and both the celiac artery and SMA branching into the expanded region (C). The celiac (yellow), SMA (pink), and right renal artery (cyan) are shown in all images. Open‐false lumens are segmented in red for (A) and (B), whereas the false lumen of (B) was entirely open and thus not segmented. Transverse US images show the celiac (top) and SMA (bottom) intersecting the aneurysm. Scale bars: 2 mm. When separated by branching arteries, total, open‐false lumen, and thrombus volume/length, animals with only the celiac artery have the largest volumes and greatest growth (D). The overall change in volume/length from day of diagnosis to day 28 was larger for the celiac only dissecting AAAs compared to the celiac and SMA group (*P ≤ 0.05). These plots represent the need to include branching vessel information when studying factors that influence dissecting AAA growth over time.

We also sought to determine if the SMA and/or celiac artery were feeding into the aneurysmal sac and whether these arteries were associated with larger volumes. Using three‐dimensional US images, we found that animals with dissecting AAAs had no branching vessels, just the celiac artery, or both the celiac artery and SMA branching into the open‐false lumen of the aneurysm. Figure 5 illustrates the three categories of aneurysms with different numbers of branching vessels feeding into the expanded region. We separated the mice based on their number of branching arteries (0, 1, or 2) and averaged total volume/length, open‐false lumen volume/length, thrombus volume/length and total change in volume/length from diagnosis day‐to‐day 28 (Fig. 5D). Total dissecting AAA volume/length was significantly larger in the celiac group compared to the group with no branching arteries (*P* < 0.05). The animals with no branching vessels had the smallest volumes (total, open‐false lumen, and thrombus), whereas the animals with just the celiac artery feeding into the aneurysm had the largest volumes and fastest growth. Animals with two branching vessels had slightly smaller volumes than those with just the celiac artery.

### Baseline ultrasound measurements predict AAA formation with logistic regression analysis

A logistic regression analysis was conducted to predict the aneurysm status (nondiseased; diseased) for mice using five variables: (1) mass, (2) suprarenal aortic diameter, (3) age, (4) circumferential strain, and (5) aortic volume/length. With the five predictors, a test of the full model against a constant only in the model was found to be statistically significant. This indicates that these predictors, as a set, significantly distinguished between disease status (chi‐square = 20.66, *P* < 0.01 with df = 5). The overall prediction success was 71% (89.8% for diseased and 40% for nondiseased). The Wald criterion, which is a chi‐squared method of evaluating significance of a single variable on a model given that the other variables included are also present in the model, demonstrated that only mass and diameter made significant contributions to predicting the disease status (*P *=* *0.022 and *P* < 0.01, respectively). The results also suggest that for every one gram increase in animal mass, the odds ratio is 1.278, and therefore, the mouse is 1.278 times more likely to become diseased with all other variables remaining constant. Similarly, for diameter, the odds ratio is 1.096, meaning that the mouse would be 1.096 times more likely to have a dissecting AAA with a 1 micrometer increase in diameter and all other variables being constant.

**Figure 6:**
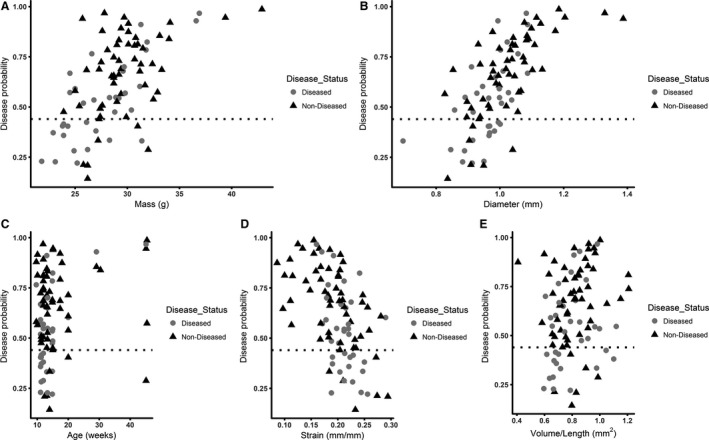
Disease probability plots for baseline metrics. Baseline mass (A), aortic diameter (B), age (C), strain (D), and total volume/length (E) for diseased and nondiseased animals are plotted with the corresponding disease probability. These data show that one metric is not sufficient to predict future disease status.

Figure 6 depicts the probability given from the logistic model that an animal will become diseased after AngII infusion plotted against the five baseline metrics, illustrating that one metric alone is not sufficient to predict future disease status. Figure 8A shows the overall performance of the logistic model. The results of this model are summarized in Table 4.

**Table 4:** Logistic regression model results; dependent variable is “diseased”

Variable	Estimate	Wald chi‐square	*P* value
Constant	−11.034	6.189	0.0128*
Mass	0.245	5.237	0.0221*
Mean diameter (mm)	7.903	6.942	00.84*
Age	−0.03	0.748	0.386
Circumferential strain (mm/mm)	−7.12	1.418	0.233
Volume/Length (mm^2^)	−1.622	0.759	0.383
Model Chi‐square = 20.666 [df = 5]		% Correct Predictions = 71%	

The Wald statistics are distributed chi‐square with 1 degree of freedom.

*Indicates that the coefficient is statistically significant at the 0.05 significance level.

### Baseline ultrasound measurements predict AAA formation with quadratic discriminant analysis

**Figure 7:**
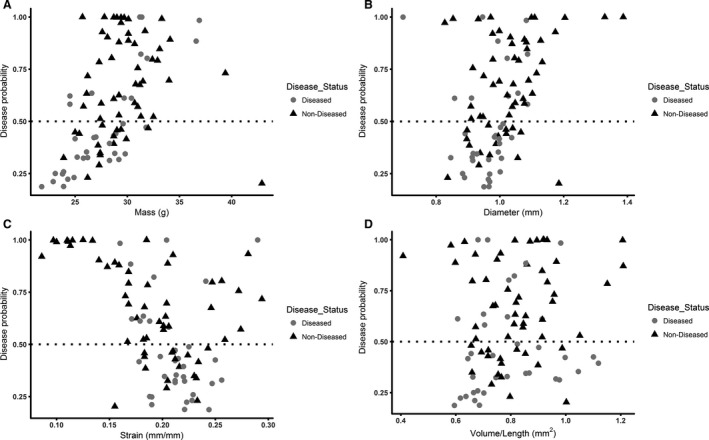
Disease probability plots for baseline metrics. Baseline mass (A), aortic diameter (B), strain (C), and volume/length (D) for diseased and nondiseased animals are plotted with the corresponding disease probability. These data show that one metric is not sufficient to predict future disease status.

**Figure 8:**
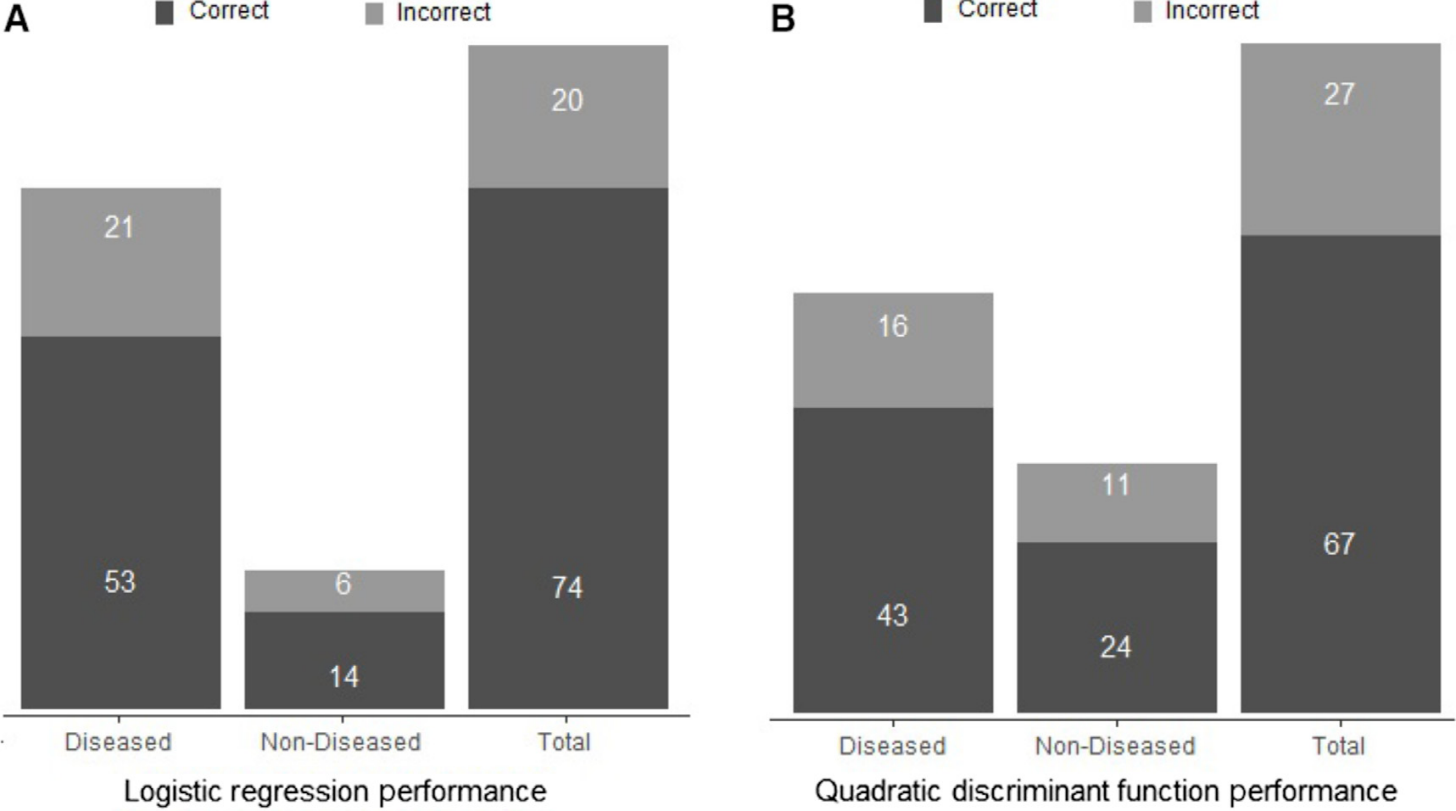
Numbers of mice predicted correctly in the diseased group, nondiseased group, and overall for the logistic regression model (A) and quadratic discriminant analysis model (B). These data highlight the ability of the prediction model to determine an animal's risk of developing a dissecting AAA or aortic rupture based only on baseline measurements.

A discriminant analysis was also used to predict the disease status for the 94 mice using six baseline variables: (1) mass, (2) age, (3) peak velocity, (4) suprarenal aortic diameter, (5) aortic volume/length, and (6) circumferential strain. The test of homogeneity of within covariance matrices was found to be significant (chi‐square = 21.60, *P *=* *0.0173 with df = 10). This means that the variance‐covariance matrices of the baseline variables were different for the group of diseased mice versus the group of nondiseased mice; therefore, a quadratic discriminant analysis was implemented on the data. The Shapiro–Wilk and Kolmogorov–Smirnov tests of normality were conducted on the baseline variables. The null hypothesis of normally distributed data was rejected only for the variable age, at the 0.05 significance level, even after applying a variety of transformations. The variable age was therefore excluded from the quadratic discriminant analysis model in order to comply with the normality assumption. The stepwise variable selection method was then implemented with a variable entry alpha level of 0.4 for screening out variables, which contribute marginally to the quadratic discriminant function. The variable peak velocity was screened out in that process. The overall prediction success for this model was 71.3% (72.3% for diseased and 68.6% for nondiseased). Figure 7 depicts the probability given from the quadratic discriminant model that an animal will become diseased after AngII infusion plotted against the four baseline metrics, again illustrating that one metric alone is not sufficient to predict future disease status. Figure 8 illustrates the overall success of both the logistic (A) and discriminant (B) models.

### Dissecting AAA diagnosis day ultrasound measurements predict final aneurysm volume

**Figure 9:**
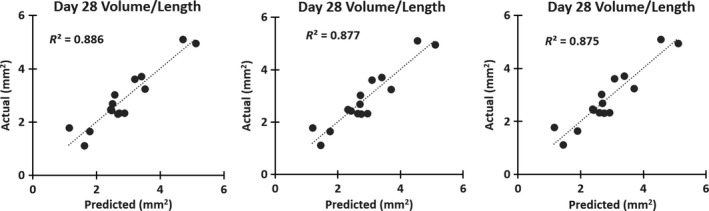
Multiple linear regression models for predicting final aneurysm size. A full 3D model including open‐false lumen and thrombus volumes (A) is comparable to a 2D model including strain (B), and a 2D model excluding strain (C) with actual volume/length at day 28 plotted against predicted values. These highly correlated regressions show that measurements taken at diagnosis day can accurately predict day 28 aneurysm volume/length.

We also created a prediction model for final (day 28) volume/length based on US measurements taken when the dissecting AAA was first observed (diagnosis day). Of the 16 mice with AAA that were tracked longitudinally, one mouse could not be included since its dissecting AAA was first observed on day 28. Six variables were used in this model, all of which were measured at diagnosis day: (1) diameter, (2) peak flow velocity, (3) circumferential strain, (4) open‐false lumen (OFL) volume, (5) thrombus volume, and (6) the number of branching vessels feeding into the aneurysm. Interaction terms were modeled between all variables (15 terms total), but the interaction between diameter and peak velocity (DPV) was the only term found to be significant and was therefore the only interaction term kept in the model. The *P*‐value for each variable was determined to show its significance for the prediction model. Strain was removed as a variable from the 3D model because it had the largest *P*‐value (0.82), demonstrating its limited effect on the model. Including only the other five variables yielded a linear prediction model with an *R* ^2^ value of 0.886 and a *P*‐value of 0.002, meaning the linear relationship is highly significant (Fig. 9A).

For a more simplified model, we investigated the effects of removing 3D measurements (OFL and thrombus volumes) from the regression. Strain was added back into this initial 2D model to determine its effects (2D_v1). We found the *R* ^2^ value for this model to be slightly decreased from the full 3D model (0.877), but the *P*‐value indicated greater significance at 0.0007 (Fig. 9B). However, the *P*‐value of strain was still large (0.740), and therefore we removed it from the model (Fig. 9C, 2D_v2). The *R* ^2^ value decreased slightly (0.875), but the *P*‐value showed greater significance than the previous two models (0.0002). Both simplified models also included the interaction term between diameter and peak velocity (DPV). The *R* ^2^ values, *P*‐values, and variables for all three models are summarized in Table 5. Multiple linear regression models for the 3D model and the 2D model without strain are shown below (Equations (3), (4)). This model suggests that we can reasonably predict day 28 aneurysm volume/length using diagnosis day measurements.

**Table 5:** Summary of variables in final dissecting AAA volume models

Model	*R* ^2^	*P*‐value	Variables Included						
			Diameter	Peak velocity	Strain	OFL	Thrombus	Branches	DPV
3D	0.886	<0.01	x	x		x	x	x	x
2D_v1	0.877	<0.01	x	x	x			x	x
2D_v2	0.875	<0.001	x	x				x	x

(3) $$\begin{array}{cc} V/L\_28_{3D}= & 8.3-\left(3.8\times {\text{diameter}}_{\mathrm{diagnosis}}\right)+\left(0.01\right.\\ & \times \text{diameter}\times \text{peakvel})-\left(\right.0.01\\ & \times {\text{peakvel}}_{\mathrm{diagnosis}})+\left(0.08\times {\text{OFL}}_{\mathrm{diagnosis}}\right)\\ & +\left(0.03\times {\text{thrombus}}_{\mathrm{diagnosis}}\right)+\left(\right.0.43\\ & \times \text{branches}) \end{array}$$

(4) $$\begin{array}{cc} V/L\_28_{2D}= & 6.3-\left(2.52\times {\text{diameter}}_{\mathrm{diagnosis}}\right)\\ & +\left(0.01\times \text{diameter}\times \text{peakvel}\right)\\ & -\left(0.01\times {\text{peakvel}}_{\mathrm{diagnosis}}\right)+\left(\right.0.44\\ & \times \text{branches}) \end{array}$$

### Histology agrees with ultrasound imaging data

In order to confirm our imaging analysis of complex dissecting AAA geometry, we performed histology on aortic tissue after 28 days. We confirmed the locations of OFL and thrombi within the false lumen, while also identifying branching arteries in a subset of animals (Fig. 2). Elastin breakage, medial dissections, adventitial collagen remodeling, and atherosclerotic plaque were also evident in multiple lesions. Taken together, the histological analysis supports the complex morphological details we identified via US.

## Discussion

The work presented here uses high‐frequency US to quantify biomechanical characteristics at baseline and longitudinally after AngII infusion with the goal of providing improved insight into aneurysm development and growth in a popular murine dissecting AAA model. We used baseline US measurements in healthy mice to create two different aneurysm formation prediction models. Additionally, US measurements acquired at the time of diagnosis could predict the final volume/length of an aneurysm using three different multiple linear regression models. While the statistical prediction models would be useful in aneurysm prevention studies, the regression model could be useful in treatment studies aimed at suppressing dissecting AAA expansion.

When differentiated by healthy and diseased, baseline mouse measurements did not show any significant difference, but there were certain trends. For example, animals that formed aneurysms or experienced aortic rupture were generally older and had greater mass, larger initial diameters and volumes, and slightly smaller circumferential strain. These trends make sense as older mice have been reported to form aneurysms more often than younger mice (Trachet et al. 2015a). It should be noted that while mice older than 24 weeks have a higher chance of AAA development or rupture, mice younger than 24 weeks have been shown to be sufficiently aged for the purposes of the model (Demetrius 2006; Trachet et al. 2015a) and are more readily available. Overall, mass of nondiseased animals appeared to be slightly less than that of animals with dissecting AAA at similar ages. For longitudinal analysis, we report significant differences in day 28 measurements between the nondiseased and diseased groups, which is to be expected given the effects of dissecting AAA (Phillips et al. 2015). However, we also reported significant increases in aortic diameter and AAA volume/length and decreases in peak flow velocity and circumferential strain between baseline and day 28 in nondiseased animals. We hypothesize that the differences in diameter and volume/length can be attributed to animal growth and the prolonged hypertensive effects of AngII. Additionally, this study showed that as diameter and volume of the vessel increased, the vessel strain and peak flow velocity decreased, likely due to the inverse relationship between both strain and blood velocity with vessel size (data not shown).

Three‐dimensional measurements make up a large portion of our aneurysm analysis and, compared to a simple diameter measurement at one location, are useful for tracking aneurysm growth over time. The ratio of volume/length is useful as a normalized metric when comparing expansion between animals due to the large variation in aneurysm lengths between animals (Goergen et al. 2010, 2011). We found that the average dissecting AAA length was 7.6 ± 2.2 mm, but these aneurysms ranged between 4.4 and 12.8 mm in length, making some type of volume normalization necessary for a true comparison between animals. The 3D measurements reported here are an important part of assessing and quantifying dissecting AAA growth and development, especially when quantifying volumes of thrombus and open‐false lumens that are reliably detected by US (Phillips et al. 2017). These volumes, in addition to branching artery information, give us different ways to characterize aneurysms and identify growth trends. Here we show a positive linear relationship between both open‐false lumen and thrombus volumes at the day of diagnosis with the final dissecting AAA volume, signifying that mice with larger initial thrombus or open‐false lumens are more prone to developing larger aneurysms by day 28. While the volumes were segmented manually, they were all acquired and quantified by a single operator. In our experience, there is not substantial inter‐operator variability when quantifying US images of these relatively large dissecting AAAs.

We also show that mice with both the celiac artery and SMA branching directly into the aneurysm have slightly smaller volumes than those animals with only the celiac artery. Though seemingly contradictory, we hypothesize that the dissecting AAAs that form with influence from both the celiac and SMA develop closer to the renal arteries. These major branching vessels may be providing additional external support, potentially restricting expansion. The length of the dissecting AAA does not seem to be a driving factor in aneurysm expansion. As others have begun to report (Trachet et al. 2015b), the effects of branching arteries on aneurysm development and growth should be considered in future studies. Specifically, focusing a study on the effects of blood flow velocities in AAAs with differing branching patterns, as others have begun to report the relationship between these factors (Phillips et al. 2017). More research is needed to determine the effects of the number, type, and size of branches on aneurysm expansion.

The prediction models presented here provide methods to both estimate the chance of aneurysm formation and estimate final aneurysm size. In both the logistic regression and quadratic linear discriminant analysis, peak velocity was found to be insignificant and therefore removed. We then utilized leave‐one‐out cross‐validation methods for both of these models to estimate the misclassification error rates (Hastie et al. 2009). Although both models exhibit similar overall prediction errors, they are different in their values of false positive and false negative rates. The quadratic discriminant analysis yields more balanced sensitivity (correctly predicting diseased) and specificity (correctly predicting nondiseased) rates of 72.88% and 68.57%, respectively. The logistic regression model, however, does well in terms of sensitivity (89.3%), but comparatively worse in terms of specificity (40.0%). We include both models here so that others may choose what is more important to their individual study. One should note, however, that the quadratic discriminant analysis could not be used in cases where any of the input predictor values violate the normality assumption.

Since mice that either develop dissecting AAA or undergo aortic rupture experience focal medial elastin breakage (Daugherty et al. 2000; Cao et al. 2010; Trachet et al. 2015b), we chose to categorize these animals together. Further delineating biomechanical differences using US or other techniques for predictively placing mice into nondiseased, dissecting AAA, and complete rupture groups is an intriguing future step. Additional studies with more animals could further separate the aortic rupture group by rupture location (arch, thoracic, suprarenal) to provide insight into the different factors influencing complete aortic rupture vs. dissection between the medial and adventitial layers. This could lead to a better understanding of biomechanical factors that influence the risk for dissecting aneurysm development and eventual rupture in humans, such as vessel diameter and wall sheer stress (Fillinger et al. 2002, 2003; Maier et al. 2010). Our final prediction model provides a simple way to estimate future growth of an aneurysm that has already formed given initial AAA measurements. Although growth rates have been categorized and forecasted in humans (Vardulaki et al. 1998), this is to the best of our knowledge the first attempt to predict aneurysm expansion in murine dissecting AAAs once diagnosed. Though we focused our work on variables measurable with in vivo US imaging to make it simpler for other groups to utilize these models, there are many other factors that could be used to enhance this predictive growth model. Blood pressure, atherosclerotic burden, and circulating biomarkers have all recently been studied (Hellenthal et al. 2009; Cao et al. 2010; Prins et al. 2014). Although circulating biomarkers could also influence dissecting AAA formation, size, and expansion, they all require repeated blood samples that are challenging in rodents with minute blood volumes. Similarly, while the hypertensive response associated with AngII has been well characterized (Goergen et al. 2011; Phillips et al. 2015, 2017), reliable blood pressure measurements are notoriously difficult to obtain in longitudinal studies. Despite these issues, inclusion of both serum biomarkers and blood pressure data in prediction models present an interesting area for future work.

The implications of this work will be impactful for others interested in evaluating dissecting AAA therapeutics by utilizing the AngII model. Due to its inherent variability (Trachet et al. 2015a), the AngII model presents two important questions: (1) will an aneurysm form, and (2) if it does, how large will it expand while the osmotic pump continues to infuse AngII. Our predictive logistic regression model could help guide others to preselect mice a priori with particular baseline metrics to ensure a higher probability of aneurysm formation, reducing the overall number of animals needed. Previous studies have reported data showing that pharmacological inhibition such as stem cell therapy and anti‐inflammatories can prevent aneurysm formation in the AngII model (Gavrila et al. 2005; Kaneko et al. 2011; Hans et al. 2012; Fu et al. 2013; Watanabe et al. 2014), but knowing the likelihood of aneurysm formation prior to pump implantation could increase the confidence in a novel therapy's effectiveness. Conversely, our predictive quadratic discriminant analysis could be useful for groups interested in studying which particular baseline metrics could accurately predict both nondiseased and diseased status. Furthermore, our longitudinal growth prediction model could be used in therapeutic AAA attenuation studies. Applying a treatment after the formation of an aneurysm is more clinically relevant than a preventative therapy (Hirsch et al. 2005; Baxter et al. 2008; Moll et al. 2011), but this approach is often underutilized due to the uncertainty of dissecting AngII AAA formation and growth. Knowing expected growth trends unique to each animal could help delineate the effects of a therapy from the inherent variability in the AngII model. Taken together, the imaging and statistical approaches described here improve our understanding of factors that affect AngII‐induced dissecting AAA formation and growth, potentially aiding in future studies focused on assessing therapeutic effectiveness.

## Conclusions

Here we show the ability of high‐frequency US to detect small biomechanical differences in the aortae of apoE^−/−^ mice infused with AngII. We further make three‐dimensional measurements of dissecting aneurysms, with a particular focus on the false lumen. These measurements, in combination with characterization of branching arteries, provide an ability to predict growth trends over time. More specifically, US data can be used to predict (1) the chance of medial elastin breakage using baseline measurements, and (2) final aneurysm volume/length given initial dissecting AAA metrics. These prediction models show promise for future therapeutic studies to more clearly differentiate the effects of both preventative and suppressive treatments from the normal variability in the AngII murine dissecting AAA model.

## Conflict of Interest

None.
